# Formamide-based production of amines by metabolically engineering *Corynebacterium glutamicum*

**DOI:** 10.1007/s00253-023-12592-3

**Published:** 2023-05-29

**Authors:** Lynn S. Schwardmann, Tong Wu, Aron K. Dransfeld, Steffen N. Lindner, Volker F. Wendisch

**Affiliations:** 1 Genetics of Prokaryotes, Faculty of Biology and CeBiTec, Bielefeld University, Universitätsstr. 25, 33615 Bielefeld, Germany; 2 Department of Biochemistry, Charité Universitätsmedizin Berlin, corporate member of Freie Universität Berlin and Humboldt-Universität Zu Berlin, Charitéplatz 1, 10117 Berlin, Germany

**Keywords:** *Corynebacterium glutamicum*, Formamide, Formamidase, Amine production, Co-cultivation

## Abstract

**Abstract:**

Formamide is rarely used as nitrogen source by microorganisms. Therefore, formamide and formamidase have been used as protection system to allow for growth under non-sterile conditions and for non-sterile production of acetoin, a product lacking nitrogen. Here, we equipped *Corynebacterium glutamicum*, a renowned workhorse for industrial amino acid production for 60 years, with formamidase from *Helicobacter pylori* 26695, enabling growth with formamide as sole nitrogen source. Thereupon, the formamide/formamidase system was exploited for efficient formamide-based production of the nitrogenous compounds L-glutamate, L-lysine, *N*-methylphenylalanine, and dipicolinic acid by transfer of the formamide/formamidase system to established producer strains. Stable isotope labeling verified the incorporation of nitrogen from formamide into biomass and the representative product L-lysine. Moreover, we showed ammonium leakage during formamidase-based access of formamide to be exploitable to support growth of formamidase-deficient *C. glutamicum* in co-cultivation and demonstrated that efficient utilization of formamide as sole nitrogen source benefitted from overexpression of formate dehydrogenase.

**Key points:**

*• C. glutamicum was engineered to access formamide.*

*• Formamide-based production of nitrogenous compounds was established.*

*• Nitrogen cross-feeding supported growth of a formamidase-negative strain.*

**Supplementary Information:**

The online version contains supplementary material available at 10.1007/s00253-023-12592-3.

## Introduction

Nitrogen is an essential main building block of life for its incorporation into numerous cellular components such as amino acids, nucleic acids, and purines (Zerkle and Mikhail 2017). Consequently, biotechnological production processes require the supplementation with an accessible nitrogen source for growth of the producer organism and biosynthesis of nitrogenous target compounds (Kampen 2014). Many bacteria, fungi, and algae naturally assimilate ammonium (NH_4_^+^) or nitrate as nitrogen source (Kampen 2014) with NH_4_^+^ as preferred nitrogen source for fast bacterial growth (Reitzer 1996). In addition, assimilation pathways for use of less common nitrogen sources have evolved, e.g., for non-peptide carbon–nitrogen nitriles (Chen et al. 2007; Duca et al. 2014; Seffernick et al. 2001). Their breakdown into NH_4_^+^ and the respective carboxylic acid is catalyzed by either nitrilase or nitrile hydratase and amidase (Willison 1993). Nitrilase activity was reported for not only filamentous fungi, yeasts, and plants, but also bacteria belonging to various genera, including *Corynebacterium* (Gong et al. 2012; Egelkamp et al. 2017). Access to non-native nitrogen sources has been attained, but these often feature unfavorable properties, which complicate efficient economic production and purification processes. Examples are *Escherichia coli* and cyanobacterial strains engineered to access melamine (Shaw et al. 2016; Selão et al. 2019), categorized as group 2B carcinogen. By contrast, formamide, the simplest naturally occurring (monocarboxylic acid) amide, shows low toxicity (Kennedy 2014). It can be considered as promising alternative nitrogen source for use in biotechnological processes, and for some bacteria even as combined nitrogen, carbon and energy source. Formamide is found in nature and in industry and is best known for its applications as solvent, catalyst, or as intermediate in the production of chemicals, mainly heterocyclic compounds, copolymers, or pharmaceuticals (Bipp and Kieczka 2011). Its market reaches an annual global size in the million ton range (Meng et al. 2022). Currently, formamide is predominantly produced from carbon monoxide and ammonia in a high temperature and pressure process (Meng et al. 2022; Bipp and Kieczka 2011).

Formamide is also ubiquitously found in the entire universe (Saladino et al. 2012), and some microbes have evolved the ability to use it as nitrogen source by hydrolysis into formate and ammonia (NH_3_), catalyzed by formamidase, an amidase with restricted substrate specificity for formamide (Hung et al. 2007). Amidases are generally widespread among bacteria and often involved in detoxification processes for the degradation of toxic amides (Newton et al. 2000; Fournand and Arnaud 2001; Liu et al. 2020), but reports on characterization of formamidases, formamide-specific amidases, are scarce (Brown et al. 1986; Skouloubris et al. 2001; Rath et al. 2010; Soriano-Maldonado et al. 2011). Today, only 14 protein structures are available and even less formamidases have been functionally characterized in detail (Mahenthiralingam et al. 1993; Parish et al. 1997; Skouloubris et al. 2001; Rath et al. 2010; Soriano-Maldonado et al. 2011), although first records of research on formamide utilization date back to 1976, when *Pseudomonas* SL-4 was described for its ability to utilize formamide as sole nitrogen, carbon, and energy source and for its involvement in the ecological carbon–nitrogen cycle (Thatcher and Weaver 1976).

Heterologous expression of the *amiF* gene from *Helicobacter pylori* in the bacteria *E. coli*, *Bacillus subtilis*, and *Klebsiella pneumoniae* enabled formamide degradation (Guo et al. 2020a, b; Ou et al. 2019). The amidase AmiF from *H. pylori* is one of the best characterized formamidases with formamide as its only identified substrate (Hung et al. 2007). *H. pylori* is known as human gastric pathogen, causing chronic gastritis and is involved in peptic ulcer disease as well as gastric carcinoma and lymphoma (Dunn et al. 1997). In that environment, NH_4_^+^ is generated by urease, AmiF, and its paralog aliphatic amidase AmiE. This primarily serves to neutralize the prevalent gastric acidity to ensure the bacterium’s survival and thriving in the human stomach (Skouloubris et al. 2001). The optimal pH for growth of *H. pylori* is at 4–6 (Dunn et al. 1997).

The model organism and well-established industrial workhorse *Corynebacterium glutamicum* is primarily used for production of nitrogenous compounds such as L-lysine since decades (Klaffl and Eikmanns 2010). Its product portfolio has been expanded to include halogenated amino acids, diamines, alkylamines, etc. (Wendisch and Lee 2020; Tsuge and Matsuzawa 2021). The Gram-positive endotoxin-free *C. glutamicum* (Srivastava and Deb 2005) is preferred over Gram-negative bacteria such as *E. coli* that often suffer from endotoxin contamination (Lee et al. 1999; Valappil et al. 2007) Its products may gain GRAS status (Wolf et al. 2021), as manifested by its deployment for the production of food-grade sugars and sugar alcohols (Hu et al. 2022), amino acids, feed, and pharmaceutical products for several decades (Leuchtenberger et al. 2005). Moreover, its advantageous high stress tolerance renders *C. glutamicum* robust for application in fermentation processes (Liu et al. 2021). In addition to its naturally broad carbon source spectrum (Zhang et al. 2020), the synthetically attained access to numerous non-native carbon sources along with its capacity to co-utilize different carbon sources (Zahoor et al. 2012; Schneider et al. 2011; Rittmann et al. 2008; Wendisch et al. 2000) make *C. glutamicum* an efficient and versatile platform organism for biotech processes.

By contrast, *C. glutamicum* shows a more restricted spectrum of nitrogen sources since it lacks secreted proteases or peptidases for extracellular degradation of nitrogenous compounds (Wendisch et al. 2022). Cultivation processes commonly rely on the provision of nitrogen in form of ammonium salts or urea (Wendisch et al. 2022). Alternatively, nitrogen can be provided in form of amino acids such as L-glutamine (Rehm et al. 2010), the key metabolite for nitrogen assimilation and transferase reactions, which is taken up by a Na^+^-dependent glutamine uptake system (Siewe et al. 1995). Another notable exception are hexosamines. While glucosamine is accessible to *C. glutamicum* wild type (WT) (Uhde et al. 2013), metabolic engineering has broadened the substrate spectrum to *N*-acetylmuramic acid (Sgobba et al. 2018a, b). Beyond, metabolic engineering has achieved the harnessing of nitrogen from monomethylamine (MMA) for the formation of the *N*-methylated amino acids sarcosine (*N*-methylglycine) (Mindt et al. 2019), *N*-methyl-L-alanine (Mindt et al. 2018), and *N*-methyl-L-phenylalanine (Kerbs et al. 2021) by overexpression of an imine reductase gene from *Pseudomonas putida* in strains overproducing the precursor oxoacid.

In this work, we extended the spectrum of nitrogen sources available to *C. glutamicum* by recruitment of a formamidase for growth and production of several target compounds with formamide as sole or combined nitrogen source. Overexpression of formate dehydrogenase gene emerged as valid tool to improve growth with formamide by counteracting toxicity of formate, the second product of formamidase.

## Material and methods

### Bacterial strains and growth conditions

All plasmids and strains used in this work are listed in Table 1.

**Table 1:** Strains and plasmids used in this work

Strain	Relevant characteristics	Source
*E. coli* DH5α	*E. coli* F-Φ80*lacZ*ΔM15 Δ(*lacZYA-argF*) U169 *endA1 recA1 hsdR17* (rK^−^, mK^+^) *supE44 thi-1 gyrA96 relA1 phoA*	Hanahan (1983)
*C. glutamicum* WT	*C. glutamicum* wild-type, ATCC13032	Kalinowski et al. (2003)
WT-EV	*C. glutamicum* WT carrying pECXT_P_syn_	Henke et al. (2021)
WT-*crimson*	*C. glutamicum* WT carrying pECXT_P_syn_-*crimson*	This work
FORM	*C. glutamicum* WT carrying pECXT_P_syn_-*amiF*	This work
FORM-*gfp*	*C. glutamicum* WT carrying pECXT_P_syn_-*amiF-gfp*_UV_	This work
FORM-Fdh_*Cg*_	*C. glutamicum* WT carrying pECXT_P_syn-_*amiF-fdh*_*Cg*_	This work
FORM-Fdh_*Ps*_	*C. glutamicum* WT carrying pECXT_P_syn-_*amiF-fdh*_*Ps*_	This work
*C. glutamicum* GRLys1Δ*sugR*Δ*ldhA*	*C. glutamicum* WTΔ*pck*, *pyc*^P458S^, *hom*^V59A^, 2 copies of *lysC*^T311I^, 2 copies of *asd*, 2 copies of *dapA*, 2 copies of *dapB*, 2 copies of *ddh*, 2 copies of *lysA*, 2 copies of *lysE*, in-frame deletion of prophages CGP1, CGP2 and CGP3, Δ*sugR*Δ*ldhA*	Pérez-García et al. (2016)
Lys-FORM	*C. glutamicum* GRLys1Δ*sugR*Δ*ldhA* carrying pECXT_P_syn_-*amiF*	This work
Lys-FORM-Fdh_*Cg*_	*C. glutamicum* GRLys1Δ*sugR*Δ*ldhA* carrying pECXT_P_syn-_*amiF-fdh*_*Cg*_	This work
Lys-FORM-Fdh_*Ps*_	*C. glutamicum* GRLys1Δ*sugR*Δ*ldhA* carrying pECXT_P_syn-_*amiF-fdh*_*Ps*_	This work
DM1729Δ*pck*	*C. glutamicum* WTΔ*pck*, *pyc*^P458S^, *hom*^V59A^Δ*pck*	Klaffl and Eikmanns (2010)
Dpa1	*C. glutamicum* DM1729Δ*pck* carrying pECXT_P_syn_-*dpaAB*	Schwardmann et al. (2022)
Dpa1-FORM	Dpa1 carrying pECXT_P_syn_-*amiF*	This work
MePhe5*	*C. glutamicum* WTΔ*qsuBCD*::P_*tuf*_-*qsuC*Δ*ppc*::P_*sod*_-*aroB*ΔP_*tkt*_::P_*tuf*_-*tkt*Δ*iolR*::P_*tuf*_-*aroE*Δt*rpEG*Δ*ilvE*Δ*aroT* carrying pEKEx3_*pheA*^FBR^ and pVWEx1_*dpkA*^P262M141L^	Kerbs et al. (2021)
MePhe5*-FORM	*C. glutamicum* MePhe5* carrying pECXT_P_syn_*-amiF*	This work
Plasmids		
pECXT_P_syn_	Tet^R^, P_trc_ *lacI*^*q*^, pGA1 *oriV*_*Cg*_, *C. glutamicum/E. coli* expression shuttle vector for constitutive expression from synthetic P_syn_ promoter	Henke et al. (2021)
pECXT_P_syn_*-amiF*	pECXT_P_syn_ derivative for constitutive expression of *amiF* of *H. pylori* 26695	This work
pECXT_P_syn_*-amiF-fdh*_*Cg*_	pECXT_P_syn_ derivative for constitutive expression of *amiF* of *H. pylori* 26695 and *fdh* of *C. glutamicum* ATCC13032 encoded by cg0616-cg0617-cg0618 in a synthetic operon	This work
pECXT_P_syn_*-amiF-fdh*_*Ps*_	pECXT_P_syn_ derivative for constitutive expression of *amiF* of *H. pylori* 26695 and *fdh* variant from *Pseudomonas* sp. 101 in a synthetic operon	This work
pECXT_P_syn_*-amiF-gfp*_UV_	pECXT_P_syn_ derivative for constitutive expression of *amiF* of *H. pylori* 26695 and *gfp*_UV_ in a synthetic operon	This work
pECXT_P_syn_-*crimson*	pECXT_P_syn_ derivative for constitutive expression of *crimson*	This work
pECXT_P_syn_*-gfp*_UV_	pECXT_P_syn_ derivative for constitutive expression of *gfp*_UV_	Henke et al. (2021)
pUC57_*amiF-ptxD*	Amp^R^, cloning plasmid with sequences of codon-optimized versions of *amiF* from *H. pylori* and *ptxD* from *Pseudomonas stutzeri* WM88	Schwardmann et al. (2022)
pVWEx1_*crimson*	Km^R^, P_tac_ *lacI*^*q*^, pHM1519, *oriV*_*Ec*_* C. glutamicum/E. coli* expression shuttle vector for inducible expression of *crimson*	Sgobba et al. (2018a, b)
pZ-ASL-FDH-v9	Amp^R^, cloning plasmid with sequence of variant of *fdh,* harboring amino acid substitutions D221Q, C255A, H379K, and S380V variant from *Pseudomonas* sp. 101, codon-optimized for *E. coli*	Calzadiaz-Ramirez et al. (2020)

*E. coli* was grown at 37 °C and 180 rpm in 50-mL baffled flasks on a rotary shaker for plasmid amplification. *C. glutamicum* was grown at 30 °C. If not indicated otherwise, precultures and production experiments of *C. glutamicum* strains were performed in 50-mL baffled flasks on a rotary shaker at 120 rpm. Growth was monitored by the measurement of optical density at 600 nm (OD_600_), using a V-1200 Spectrophotometer (VWR, Radnor, PA, USA). OD_600_ was converted into cell dry weight concentrations (CDW) according to a previously determined factor of an OD600 of 1 to correspond to 0.25 g L^−1^ CDW.

Routinely, growth experiments of *C. glutamicum* strains were performed in a BioLector microcultivation system (m2p-labs, Aachen, Germany) in volumes of 1 mL in a 48-well flower plate at 85% humidity and 1100 rpm. Growth was followed using the backscatter light signal at 620 nm, given in arbitrary units (a.u.).

Precultures were inoculated from fresh lysogeny broth (LB) agar plates and grown in LB or brain–heart infusion broth (BHI) (ROTH, Karlsruhe, Germany) overnight. Main cultures for growth or production experiments were inoculated to an OD_600_ of 1, using overnight precultures, which were washed and resuspended in CgXII minimal medium without accessible nitrogen (N-CgXII, lacking the nitrogen sources urea and (NH_4_)_2_SO_4_ as compared to CgXII minimal medium) (Eggeling and Bott 2005). Routinely, cultivations were performed in CgXII minimal medium or N-CgXII, supplemented the indicated concentrations of nitrogen in form of urea and (NH_4_)_2_SO_4_ or formamide with 40 g L^−1^ glucose as carbon source. Indicated nitrogen concentrations in form of urea and (NH_4_)_2_SO_4_ correspond to their ratio in standard CgXII minimal medium. Deviating, MePhe5*-FORM was grown with 20 g L^−1^ glucose and supplemented with 0.35 M monomethylamine (MMA), 0.2 mM L-tryptophan, and 0.8 mM L-phenylalanine, L-isoleucine, and L-leucine, respectively (Kerbs et al. 2021).

When appropriate, precultures of *E. coli* strains were supplemented with 10 µg mL^−1^ tetracycline, 50 µg mL^−1^ kanamycin, or 100 µg mL^−1^ ampicillin and pre- and main cultures of *C. glutamicum* with 5 µg mL^−1^ tetracycline, 25 µg mL^−1^ kanamycin, and 100 µg mL^−1^ spectinomycin. For the induction of the expression plasmids pVWEx1 and pEKEx3 in main cultures, 1 mM Isopropyl-β-D-1-thiogalactopyranoside (IPTG) was added. Glutamate production was elicited by exposure to 8 µg mL ^−1^ ciprofloxacin during mid-exponential growth, when an OD_600_ of 9 was reached (Lubitz and Wendisch 2016).

### Genetic engineering for plasmid and strain construction

All listed oligonucleotides for DNA amplification and sequencing (Supplemental Tab. S1) were purchased from Metabion (Planegg/Steinkirchen, Germany) or Sigma-Aldrich (Ulm, Germany). All commercially available kits and enzymes were used according to the suppliers` instructions. Plasmids and DNA were isolated and purified using the GeneJET plasmid miniprep kit (Thermo Fisher Scientific, Schwerte, Germany) and the NucleoSpin® Gel and PCR Clean-up kit (MACHEREY–NAGEL GmbH & Co. KG, Düren, Germany). Genomic DNA of *C. glutamicum* was isolated by salt precipitation (Eikmanns et al. 1994). DNA concentrations were measured at 260 nm by a V-1200 Spectrophotometer (VWR, Radnor, PA, USA).

Genes were amplified from plasmids or genomic DNA of *C. glutamicum* ATCC13032 with the respective oligonucleotides (Supplemental Tab. S1) by ALLin™ HiFi DNA Polymerase (highQu GmbH, Kraichtal, Germany). Primer overhangs were used to insert a consensus ribosome binding site (RBS) sequence (GAAAGGAGGCCCTTCAG) in front of the genes *amiF*, *gfp*_UV_, *crimson*, and *fdh*_*Ps*_, and an optimized RBS (CTGAAGGGCCTCCTTTC, designed using the Salislab software (Cetnar and Salis 2021)) was inserted in front of the gene *fdh*_*Cg*_*.*

The plasmids pECXT_P_syn_ and pECXT_P_syn_-*amiF* were linearized by restriction with *BamHI* or *XbaI*, respectively (New England Biolabs, Frankfurt, Germany), followed by dephosphorylation (Antarctic phosphatase, New England Biolabs, Frankfurt, Germany). Plasmids were assembled via the method of Gibson (Gibson et al. 2009). Newly constructed expression plasmids were sequenced with respective oligonucleotides (Supplemental Tab. S1) for verification of correct assembly and sequence validity of inserted genes.

Standard molecular genetic techniques followed previously described procedures (Sambrook et al. 1989). The CaCl2 method was applied to prepare chemocompetent *E. coli* cells for transformation by heat shock at 42 °C (Sambrook et al. 1989). Electrocompetent *C. glutamicum* cells were transformed by electroporation, followed by a heat shock at 46 °C (van der Rest et al. 1999). Recombinant clones were confirmed by colony PCR.

### Stable isotope labeling

For isotope-tracing of nitrogen, 60 mM nitrogen in the form of 15^N^ labeled (NH_4_)_2_SO_4_ and/or formamide (Sigma-Aldrich, Ulm, Germany) and/or respective unlabeled substrates were provided as nitrogen sources for cultivation in N-CgXII. Cells were grown in a BioLector microcultivation system for 48 h and harvested by centrifugation. For analysis of lysine production, supernatants were immediately stored at − 20 °C, while cell pellets were washed and resuspended in H_2_O prior to storage at − 20 °C until analysis.

Proteins were hydrolyzed at 95 °C for 24 h using 6 M HCl (You et al. 2012), which was removed by evaporation under stream of N_2_ at 95°. Samples were resuspended in H_2_O and centrifuged for the removal of insoluble compounds for analysis of the supernatants by UPLC-ESI–MS (Giavalisco et al. 2011). Amino acid mass analysis was performed with a Waters Acquity UPLC system (Waters, Eschborn, Germany) equipped with a HSS T3 C18 reversed-phase column (100 mm × 2.1 mm, 1.8 µm; Waters, Eschborn, Germany), using 0.1% formic acid in H_2_O (A) and 0.1% formic acid in acetonitrile (B) at a flow rate of 0.4 mL min^−1^ and the following gradient: 0–1 min 99% A; 1–5 min linear gradient 99 to 82% A; 5–6 min linear gradient 82 to 1% A; 6–8 min 1% A; 8–8.5 min linear gradient 1 to 99% A; and 8.5–11 min re-equilibration.

An exactive mass spectrometer (Thermo Fisher Scientific, Schwerte, Germany) was used in positive ionization mode at a scan range of 50.0–300.0 m/*z* for acquisition of mass spectra, and the Xcalibur™ software (Thermo Fisher Scientific, Schwerte, Germany) was used for data analysis. Retention times were determined by analyzing amino-acid standards (Sigma-Aldrich) under the same conditions.

### Assay for ammonium detection

For the measurement of NH_4_^+^, cells were grown in N-CgXII, supplemented with 60 mM nitrogen in form of urea and (NH_4_)_2_SO_4_ or formamide for 24 h. Samples were harvested by centrifugation and the NH_4_^+^content of the supernatants was quantified with an ammonia assay kit (Megazyme, Bray, Ireland).

### Enzyme activity assay for formamidase AmiF

Crude extracts were gained from cells, grown in 50 mL LB in shake flasks overnight and harvested by centrifugation (3100 g, 7 min) at 4 °C. All following steps were performed at 4 °C. Cells were washed 3 times in assay buffer (50 mM phosphate, pH 7), resuspended in 5 mL and lysed by sonication (UP 200S, Dr. Hielscher GmbH, Teltow, Germany), applying ultrasound at an amplitude of 60% and a duty cycle of 0.5 s for 9 min. The protein containing supernatant was attained by centrifugation (25,000 g, 1 h), and the total protein concentrations of the crude extracts were analyzed by the method of Bradford, using bovine serum standard as reference (Bradford 1976).

Formamidase activity was measured by means of formamide hydrolysis, which was detected by the formation of free NH_4_^+^ as described previously (Ou et al. 2019; Weatherburn 1967). The reaction was carried out in 1 mL assay buffer, containing 2.5 mg L^−1^ total crude extract protein, started by the addition of 200 mM formamide, and run for 1 h at 30 °C. The dying was implemented as described elsewhere (Weatherburn 1967), but incubated for 35 min. One unit (U) of formamidase activity was defined as the quantity of total protein required to deplete 1 μM formamide min^−1^.

### Fluorescence analysis by flow cytometry

The cellular composition of co-cultures was analyzed by means of fluorescence using flow cytometry (flow cytometer Gallios™, Beckman Coulter, Krefeld, Germany). Samples of co-cultures were taken after 24 h and diluted to an OD_600_ of 0.1 in N-CgXII for immediate analysis of 20 000 cells per sample. Fluorescence was excited at 405 nm by a blue solid-state laser for monitoring of the forward- (FSC) and side-scatter (SSC) signals. The Gfp_UV_ and Crimson signals were detected via 525/50 nm and 660/20 nm band-pass filters, respectively. WT-EV cells served as reference for preliminary adjustment for autofluorescence.

### Product quantification by HPLC analysis

Extracellular DPA and amino acids glutamate, lysine, and NMePhe were quantified by use of a high-pressure liquid chromatography system (HPLC) (1200 series, Agilent Technologies Deutschland GmbH, Böblingen, Germany). Cells were grown in N-CgXII, supplemented with 60 mM nitrogen in form of urea and (NH_4_)_2_SO_4_ or formamide as indicated. Samples were taken after 48 or 72 h and centrifuged for the storage of supernatants at − 20 °C until analysis.

Amino acids were separated by reversed phase HPLC with a pre- (LiChrospher 100 RP18 EC-5µ (40 × 4 mm), CS Chromatographie Service, Langerwehe, Germany), and a main-column (LiChrospher 100 RP18 EC-5µ, 125 × 4.6 mm, CS Chromatographie Service, Langerwehe, Germany) and detected by a fluorescence detector (FLD G1321A, 1200 series, Agilent Technologies, Böblingen, Germany).

For detection of lysine and glutamate, samples were derivatized with ortho-phthaldialdehyde (OPA). Analysis followed previously described procedures with asparagine as internal standard (Schneider and Wendisch 2010). Compounds were detected at 230 nm excitation and 450 nm emission wavelengths. For detection of NMePhe, samples were derivatized with fluorenylmethyl chloroformate (FMOC) (Karl Roth, Karlsruhe, Germany)(Schneider et al. 2012). Compounds were separated at a flow rate of 1.2 mL min^−1^ with sodium acetate (50 mM, pH 4.2) (A) and acetonitrile (B) as eluents, applying the following gradient: 0 min 38% B, 5 min 38% B, 10 min 48% B, 12 min 52% B, 13 min 57% B, 14 min 63% B, 16 min 68, 17 min 76% B and 20 min 38%. L-proline served as internal standard, and fluorescence was detected at 250 nm excitation and 410 nm emission wavelengths (Kerbs et al. 2021). DPA was separated with an amino exchange column (Aminex, 300 × 8 mm, 10 µm particle size, 25 Å pore diameter, CS Chromatographie Service, Langerwehe, Germany) under isocratic conditions, using 5 mM H_2_SO_4_ as mobile phase at a flow rate of 0.8 mL min^−1^ for 30 min and detected by the refractive index signal (RID G1362A, 1200 series, Agilent Technologies, Böblingen, Germany) as described previously (Schwardmann et al. 2022).

## Results

### Assessment of the suitability of *C. glutamicum* for formamide utilization

Formamide is an organic nitrogen source that is degraded by formamidase to yield formate and NH_4_^+^. To determine if formamide or formate inhibit growth of *C. glutamicum* in minimal medium, the empty vector control carrying strain *C. glutamicum* ATCC13032 (named WT-EV) was grown in CgXII minimal medium in the presence of 0–160 mM formamide or sodium formate.

**Fig. 1:**
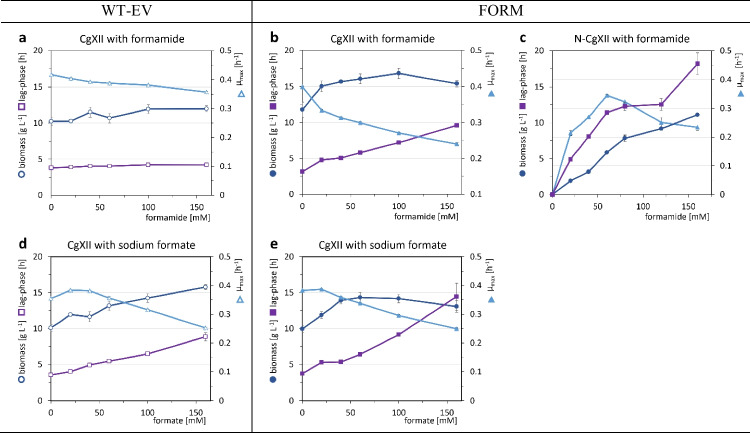
Influence of formamide and formate on the growth of *C. glutamicum* strains. Biomass (dark blue circles), *µ*_max_ (light blue triangles), and lag phase (purple squares) of strains WT-EV (open symbols) and FORM (closed symbols), cultivated in CgXII minimal medium (**a**, **b**, **d**, **e**) or N-CgXII (**c**) supplemented with 40 g L^−1^ glucose and either 0–160 mM formamide (**a**, **b**, **c**) or 0–160 mM sodium formate (**d, e**) in a BioLector microcultivation system for 96 h. Values represent means with standard deviations of triplicate cultivations

The exposure to increasing formamide concentrations from 0 to 160 mM had only a minor effect on biomass formation of strain WT-EV, but slightly decreased the maximal specific growth rate *µ*_max_ by up to 14% from 0.42 ± 0.00 to 0.36 ± 0.00 h^−1^ (Fig. 1a). A reduction of the growth rate to half maximal was calculated to be reached at a concentration of 690 mM, which exceeds the routinely used nitrogen content of 468 mM in form of urea and (NH_4_)_2_SO_4_ in CgXII minimal medium and demonstrates high tolerance. The presence of sodium formate is known to inhibit growth of *C. glutamicum* (Witthoff et al. 2012; Ryabchenko et al. 2020). When cultivated in CgXII minimal medium in the presence of 0–160 mM sodium formate, the growth rate was reduced to half-maximal at 227 ± 3 mM formate (Fig. 1d) and lag phase duration increased proportionally to the formate concentration (Fig. 1d). By contrast, this was not observed for addition of 160 mM sodium ions as Na_2_SO_4_ salt (data not shown). The presence of formate increased the maximal biomass concentration, probably due to NADH formation as consequence of its oxidation to carbon dioxide by formate dehydrogenase. In conclusion, the high tolerance of *C. glutamicum* towards formamide provides a promising basis for its use as amine source by engineered strains.

### Establishment of formamide assimilation by engineered *C. glutamicum*

Inspection of the *C. glutamicum* ATCC13032 genome did not identify homologs of AmiF. Additionally, no growth was observed when incubating *C. glutamicum* with formamide as sole nitrogen (N) source. Hence, with the goal to make formamide an accessible nitrogen source for *C. glutamicum* WT, it was equipped with a plasmid for constitutive expression of codon optimized version of the gene *amiF*, coding for formamidase from *H. pylori* 26695, resulting in strain *C.glutamicum* WT(pECXT_P_syn_-*amiF*), from here on referred to as FORM.

First, formamidase activity was analyzed in crude extracts of strains FORM and the empty vector control strain WT-EV, obtained after growth in LB by conducting an enzymatic activity assay. The detection of 6.0 ± 0.8 U mg_total_ p_rotein_^−1^ in crude extract of strain FORM*,* but no detectable activity (< 0.4 U mg^−1^) for the empty vector carrying control strain WT-EV (Fig. 1a) demonstrated functional expression of *amiF* in *C. glutamicum*.

**Fig. 2:**
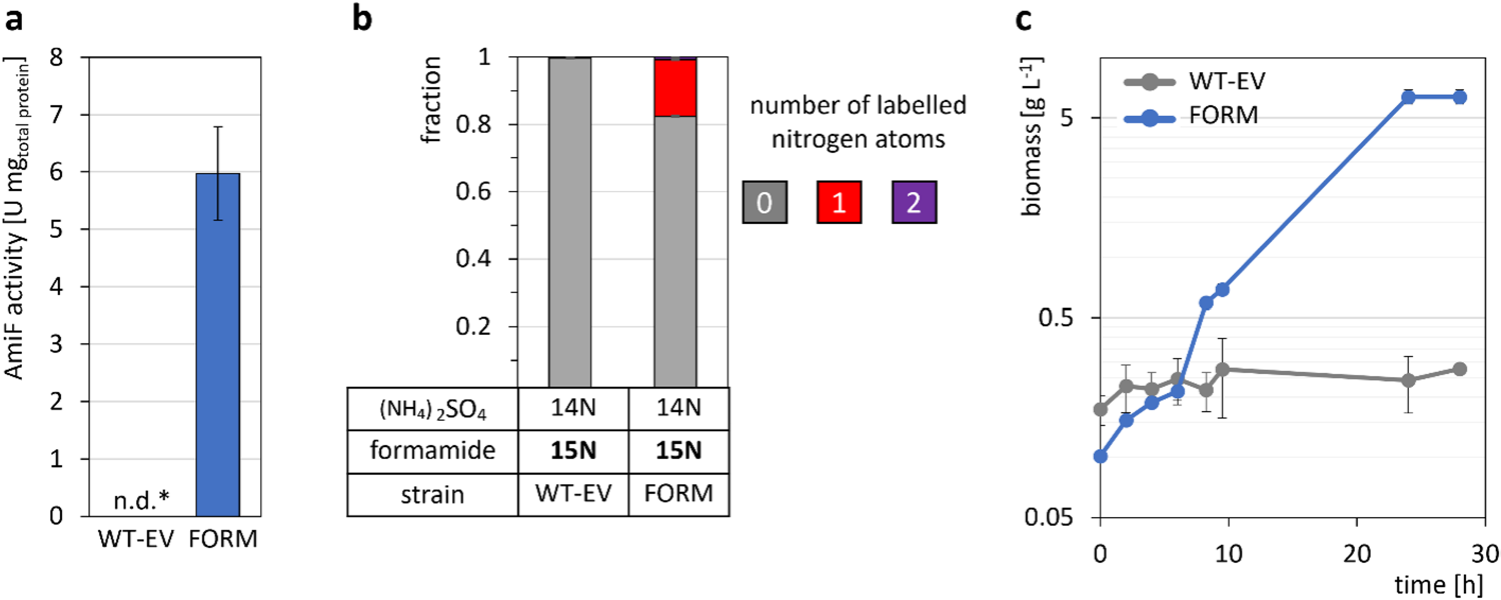
AmiF activity in crude extracts (**a**), ^15^N labeling of L-lysine from ^15^N-labeled formamide (**b**), and growth (**c**) of formamidase-expressing strain FORM (blue) and WT-EV (grey) with formamide as sole nitrogen source. Cells were grown in LB over night for crude extract preparation (**a**). Cells were grown in CgXII minimal medium containing 468 mM unlabeled (14N) N in form of urea and (NH_4_)_2_SO_4_, supplemented with 60 mM ^15^N labeled formamide (15N) and 40 g L^−1^ glucose, in a BioLector microcultivation system for 24 h. The patterns depict the fractions of 0 (grey), 1 (red), or 2 (purple) labeled nitrogen atoms per molecule of lysine in the biomass. Standard deviations refer to triplicate cultivations. For growth assessment, cells were grown in N-CgXII minimal medium, supplemented with 40 mM formamide for 28 h (**c**). Values represent means with standard deviations of triplicate measurements or cultivations. *n.d., not detectable (< 0.4 U mg.^−1^)

Next, we grew both strains in regular CgXII minimal medium containing urea and (NH_4_)_2_SO_4_ as nitrogen sources with added ^15^N-labeled formamide. When 60 mM labeled (^15^N) formamide was provided in CgXII minimal medium in addition to 468 mM of N in form of unlabeled (^14^N) urea and (NH_4_)_2_SO_4_, 17% of lysine molecules of strain FORM were labeled once, but lysine of WT-EV lacked detectable ^15^N-labeling (Fig. 2b). This revealed that the ammonium from formamide was only accessible for strain FORM, even at abundant availability of natively accessible nitrogen (Fig. 2b).

Additionally, strains WT-EV and FORM were cultivated in CgXII minimal medium without nitrogen in form of urea or (NH_4_)_2_SO_4_ (N-CgXII), supplemented with 40 mM formamide as sole potential nitrogen source to evaluate the capacity of AmiF activity to support growth with formamide as sole nitrogen source. A formamide concentration of 40 mM allowed to monitor growth with hardly any inhibitory effects due to formamide or formate. While WT-EV was unable to grow in N-CgXII with formamide, formamide supported growth of strain FORM to a maximal biomass concentration of 6.3 ± 0.5 g L^−1^ with a maximal growth rate of 0.17 ± 0.01 h^−1^ (Fig. 2c). When strain FORM was grown with 40 mM nitrogen in form of urea and (NH_4_)_2_SO_4_, growth to a biomass concentration of 5.2 ± 0.1 g L^−1^ was faster (0.28 ± 0.02 h^−1^) than with formamide (data not shown).

**Fig. 3:**
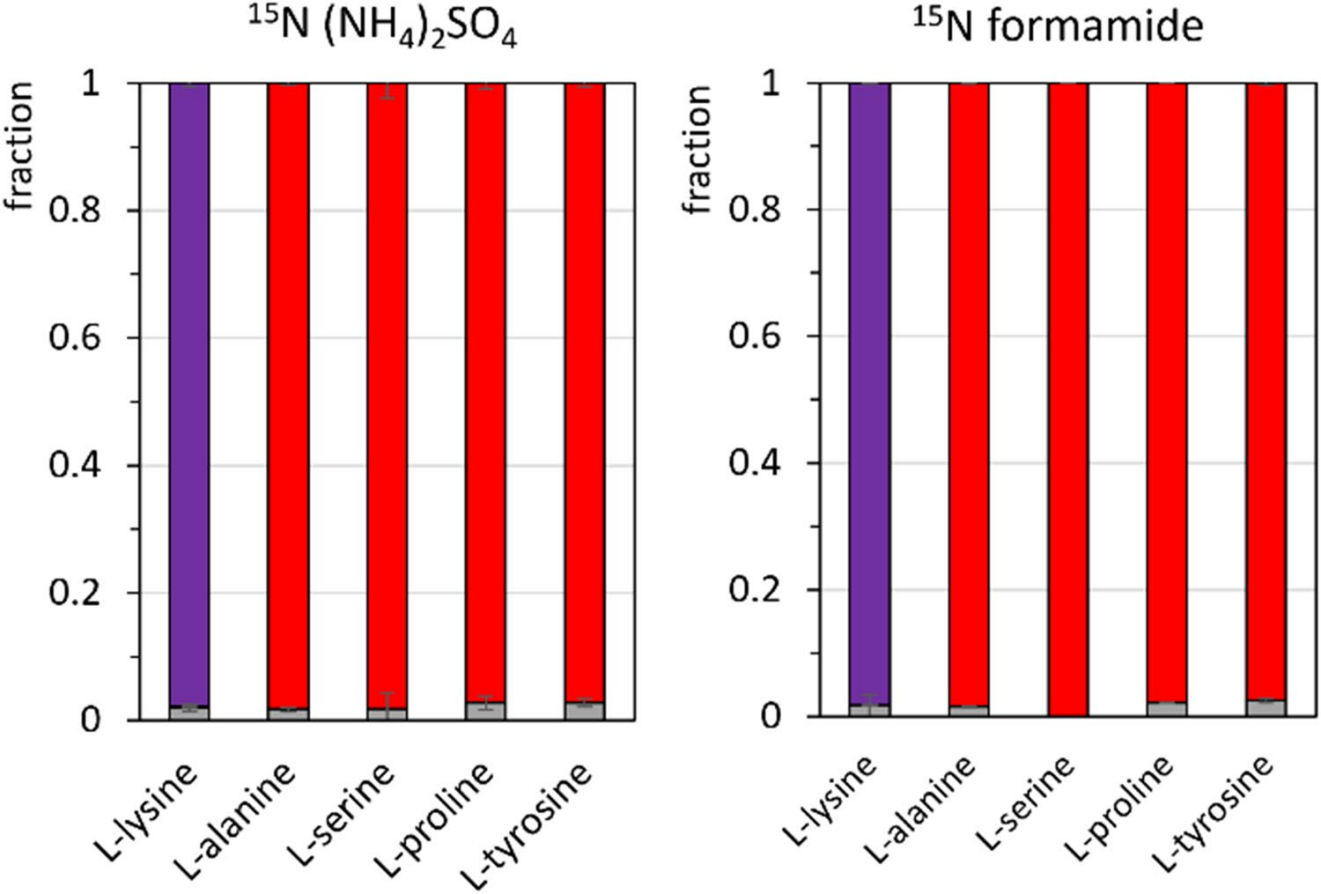
^15^N labeling of L-lysine, L-alanine, L-serine, L-proline, and L-tyrosine in strain FORM. Cells were grown in N-CgXII, supplemented with 60 mM ^15^N-labeled (NH_4_)_2_SO_4_ or formamide and 40 g L^−1^ glucose, in a BioLector microcultivation system for 24 h. The patterns depict the fractions of 0 (grey), 1 (red), or 2 (purple) labeled nitrogen atoms per molecule of lysine in the biomass. Standard deviations refer to triplicate cultivations

Finally, both strains were cultivated in N-CgXII minimal medium supplemented with 60 mM unlabeled or ^15^N-labeled ammonium sulfate and/or formamide as nitrogen sources and the labeling pattern in the biomass was measured for the five representative proteinogenic amino acids L-lysine, L-alanine, L-serine, L-proline, and L-tyrosine. When strain FORM was cultivated with ^15^N-labeled ammonium sulfate or ^15^N-labeled formamide, almost complete labeling of the single nitrogen atoms was observed for alanine, serine, proline, and tyrosine, while both nitrogen atoms of lysine were labeled (Fig. 3).

**Fig. 4:**
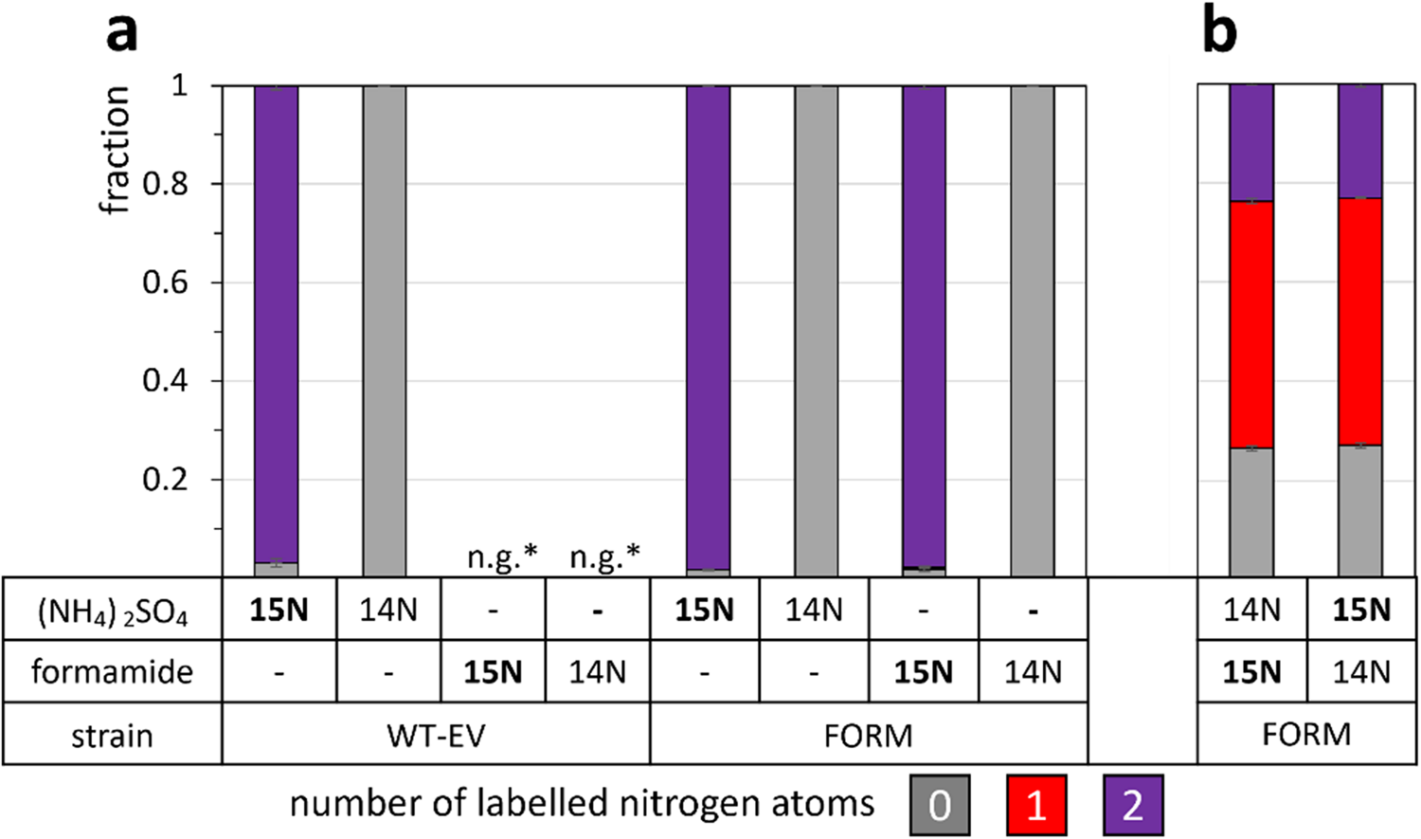
^15^N labeling of lysine in WT-EV and FORM from ^15^N-labeled ammonium sulfate or formamide. Cells were grown in N-CgXII, supplemented with 60 mM unlabeled (^1^14N) or labeled (15N) (NH_4_)_2_SO_4_ or formamide (**a**), or with 30 mM labeled or unlabeled (NH_4_)_2_SO_4_ and formamide, respectively (**b**), and 40 g L^−1^ glucose, in a BioLector microcultivation system for 24 h. The patterns depict the fractions of 0 (grey), 1 (red), or 2 (purple) labeled nitrogen atoms per molecule of lysine in the biomass. Standard deviations refer to triplicate cultivations. *n.g., no growth with formamide

Both strains showed double ^15^N-labeled lysine with ^15^N-labeled (NH_4_)_2_SO_4_, but lysine was unlabeled when unlabeled (NH_4_)_2_SO_4_ was provided (Fig. 4a). Strain WT-EV was unable to utilize formamide, but provision of ^15^N-labeled formamide as sole nitrogen source to strain FORM allowed growth and resulted in double ^15^N-labeled lysine (Fig. 4a). This demonstrated that strain FORM assimilated formamide as sole nitrogen source as well as ammonium sulfate. This notion was further supported when growth of *C. glutamicum* FORM with a mixture of 30 mM ^15^N-labeled formamide and unlabeled ammonium sulfate, respectively, was compared to growth with a mixture of unlabeled formamide and ^15^N-labeled ammonium sulfate since the labeling patterns of lysine were comparable under both conditions (Fig. 4b).

Taken together, we have shown that the engineered *C. glutamicum* strain FORM efficiently utilizes formamide as sole or combined nitrogen source to support growth.

### Co-culturing formamidase-positive with formamidase-negative strains

**Fig. 5:**
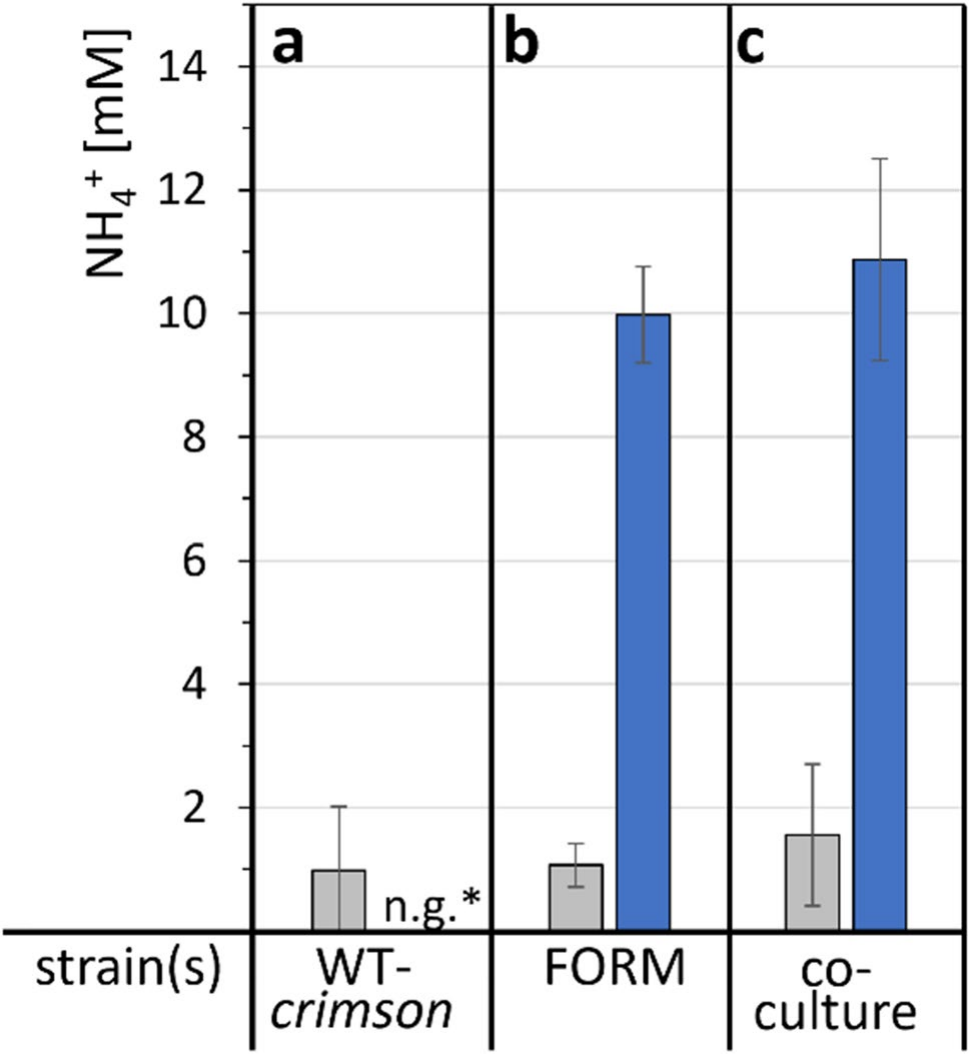
Free ammonium in supernatants of cells of WT*-crimson* (**a**) and FORM-*gfp* (**b**). Cells were either grown separately (**a**, **b**) or in co-culture inoculated with 70% WT*-crimson* and 30% FORM-*gfp* (**c**), supplemented with 60 mM N in form of urea and (NH_4_)_2_SO_4_ (grey) or with 60 mM formamide (blue) and 40 g L^−1^ glucose as carbon source, for 24 h. Values represent means of triplicate measurements with standard deviations. *n.g., no growth with formamide

Since formamidase hydrolyzes formamide to yield formate and ammonium, we tested if ammonium leakage out of the cell was exploitable in a co-culture approach using a formamidase-positive and a formamidase-deficient strain. To allow distinction of the used strains by fluorescence microscopy and FACS analysis, the fluorescence reporter Crimson was constitutively expressed in formamidase-negative *C. glutamicum* WT-EV, while GFP was expressed as part of a synthetic operon with *amiF* in strain FORM, resulting in strains WT-*crimson* and FORM-*gfp*, respectively. To test if the formamidase-positive strain FORM-*gfp* can provide ammonium from formamide as sole nitrogen source in the growth medium not only for itself, but also for formamidase-negative strain WT-*crimson* by ammonium export or leakage, supernatants of FORM-*gfp* grown with 60 mM N either provided as urea and (NH_4_)_2_SO_4_ or as formamide were analyzed for NH_4_^+^. With urea and (NH_4_)_2_SO_4_, less than 2 mM NH_4_^+^ were detected (Fig. 5b), while 10.0 ± 0.8 mM NH_4_^+^ was detected in the supernatants of strain FORM-*gfp* grown with formamide (Fig. 5b).

**Fig. 6:**
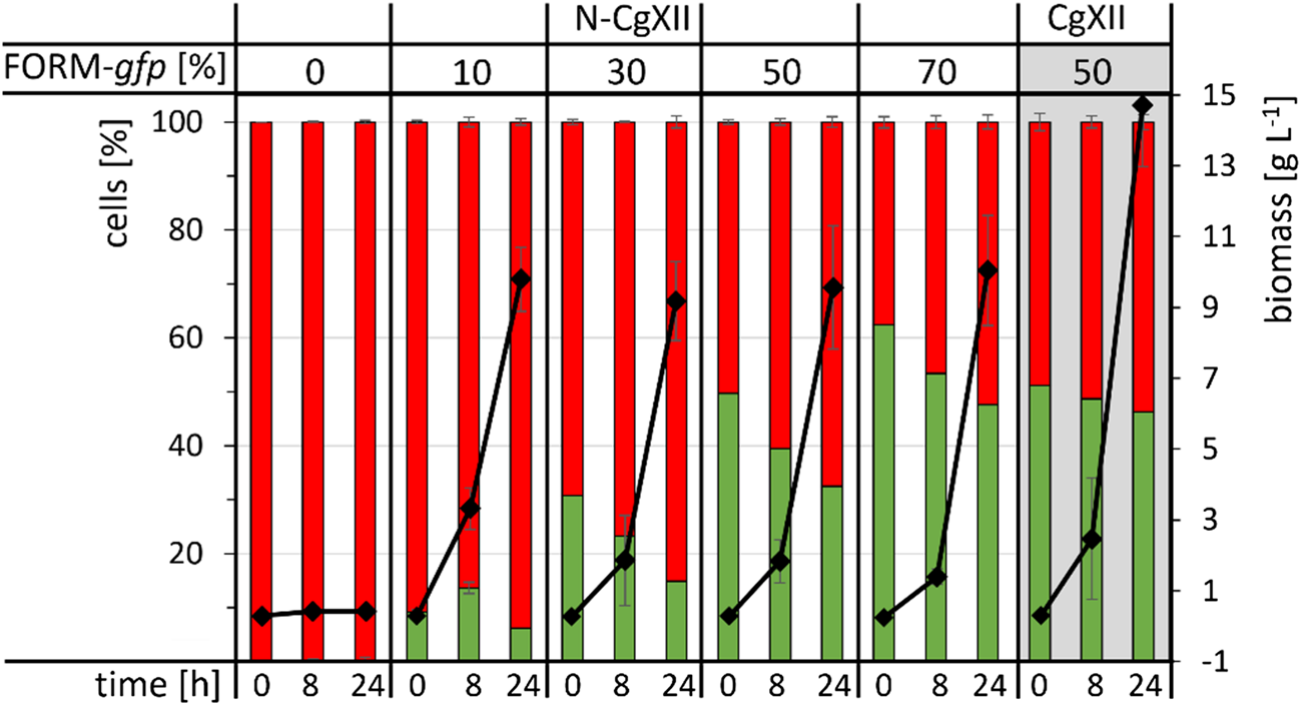
Co-cultures of strains WT-*crimson* (red) and FORM*-gfp* (green) in varied inoculum ratios. To test if FORM-*gfp* can provide nitrogen from formamide for growth of *amiF*-deficient WT-*crimson*, cells were grown with 60 mM formamide as sole nitrogen source in N-CgXII, whereas a control cultivation in CgXII minimal medium supplemented with 60 mM N in form of urea and (NH_4_)_2_SO_4_ was also made (grey background). 40 g L^-1^ glucose was added as carbon source. The inoculum contained the indicated percentage of FORM-*gfp* with the rest to 100% being WT-*crimson* cells. Biomass formation (black diamond) and culture compositions (stacked red and green bars) were determined after 0, 8, and 24 h by OD_600_ and FACS analysis. Values represent means with standard deviations of triplicate cultivations

Next, a co-cultivation of formamidase-negative WT-*crimson* and formamidase-positive FORM-*gfp* was performed, and the inoculum ratio was varied to contain 0, 10, 30, 50, or 70% of FORM-*gfp* (with 100, 90, 70, 50, or 30% WT-*crimson*, respectively). In CgXII minimal medium containing 60 mM N as urea and (NH_4_)_2_SO_4_, both strains grew from 0.25 to 15 g L^−1^ biomass in 24 h and roughly maintained a ratio of 50%/50% throughout cultivation (Fig. 6). In the formamide containing medium N-CgXII, WT-*crimson* alone (0% FORM-*gfp)* could not grow as expected (Fig. 6). Remarkably, all co-cultivations of WT-*crimson* with FORM-*gfp* resulted in growth of both strains to combined biomass concentrations of about 10 g L^−1^ (Fig. 6). This indicated that nitrogen released by cleavage of formamide to formate and NH_4_^+^ supported growth of formamidase-negative WT-*crimson*. For example, in a co-cultivation inoculated with 30% of FORM-*gfp* cells and 70% WT-*crimson* cells to a combined biomass concentration of about 0.25 g L^−1^, the biomass after 24 h consisted of about 1.5 g L^−1^ FORM-*gfp* cells and 8.5 g L^−1^ WT-*crimson* cells (Fig. 6). Similar to the single cultivation of FORM-*gfp*, supernatants of this co-cultivation showed about 11 mM of extracellular free ammonium (Fig. 5c). Thus, FORM-*gfp* grown with formamide produced sufficient ammonium from formamide to support growth of a strain unable to use formamide.

### Establishing L-lysine overproduction with formamide as sole nitrogen source

The overproduction of nitrogenous compounds such as amino acids, in particular lysine that contains two nitrogen atoms, requires higher nitrogen concentrations in the growth medium. Since we observed slight growth inhibition of the formamidase-negative wild type-derived strain (Fig. 1a), it was tested first, if formamide affects the formamidase-positive strain FORM differently than a formamidase-negative strain. Growth experiments with strain FORM in CgXII containing 0, 20, 40, 60, 80, 120, or 160 mM formamide in addition to the regular ammonium and urea concentrations showed roughly similar effects on the growth rate, lag phase, and biomass concentration as observed for the formamidase-negative strain WT-EV (Fig. 1b). When formamide was used as sole nitrogen source in the growth medium N-CgXII, FORM grew with the highest growth rate at 60 mM formamide, which complies with the reported affinity of AmiF for formamide (*K*_*m*_ = 32 ± 8.7 mM; Skouloubris et al. 2001). The growth rate was reduced to half-maximal at about 200 mM (Fig. 1b, c). However, the use of formamide as sole nitrogen source increased the lag phases with increasing formamide concentrations to a larger extent than as combined nitrogen source (Fig. 1b, c). By contrast, although growth was slowed, the attained maximal biomass concentrations increased with increasing formamide concentrations from 20 to 160 mM (Fig. 1c). In sum, our observations emphasize a clear tradeoff between immediate fast growth and high biomass formation with formamide as sole nitrogen source. To compromise for sufficient nitrogen provision to allow high biomass formation at an adequate growth rate, all following cultivations were performed using 60 mM formamide.

As it is known that formate, the second product of the formamidase reaction besides NH_4_^+^, has an inhibitory effect on growth of *C. glutamicum* (Witthoff et al. 2012; Ryabchenko et al. 2020), we compared its growth inhibitory effect for formamidase-negative strain WT-EV and formamidase-positive strain FORM. The effect of formate on the growth rate was comparable between the two strains (Fig. 1d, e); however, lag phases were longer for strain FORM. Moreover, a positive effect of formate on the biomass concentration of strain FORM was observed only until 40 mM formate, when a plateau was reached, while the formate concentration correlated with increased biomass formation by strain WT-EV (Fig. 1d, e). Thus, higher formate concentrations are tolerated less when formamidase gene *amiF* is expressed.

Formate is oxidized to carbon dioxide by formate dehydrogenase with concomitant formation of a reduced redox cofactor. Endogenous formate dehydrogenase Fdh_*Cg*_ is NAD^+^-dependent, while an NADP^+^-dependent variant of *Pseudomonas* sp. 101 (Fdh_*Ps*_) has been described (Calzadiaz-Ramirez et al. 2020). Both genes were overexpressed in strain FORM as part of a synthetic operon with *amiF*, yielding strains FORM-Fdh_*Cg*_ and FORM-Fdh_*Ps*._

**Fig. 7:**
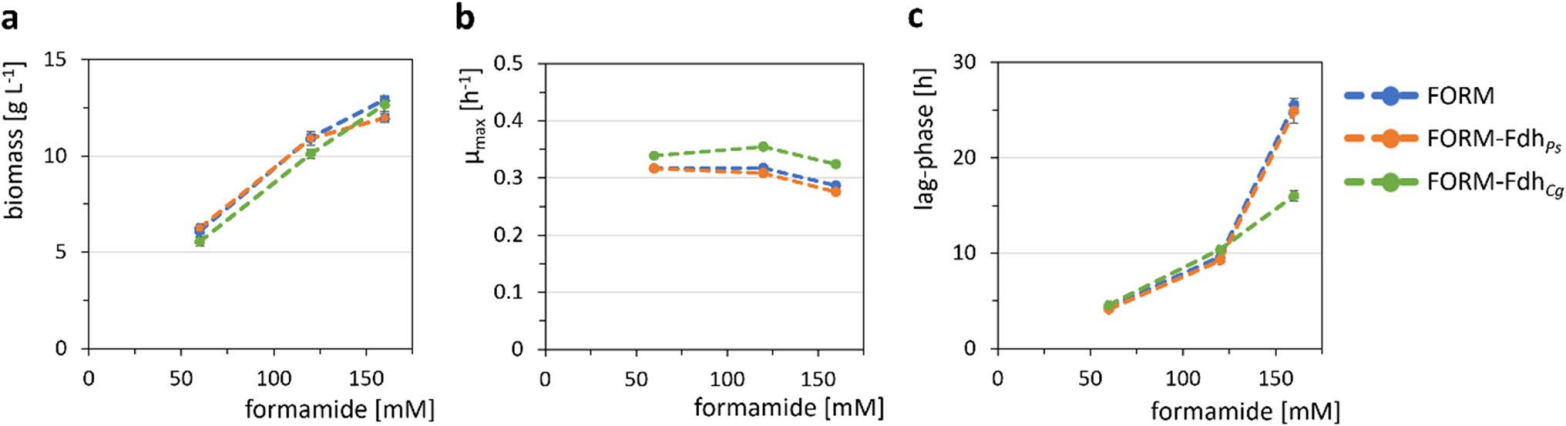
Biomass formation (**a**), maximal growth rates *µ*_max_ (**b**), and lag phases (**c**) of FORM (blue) and formate dehydrogenase overexpressing strains FORM-Fdh_*Cg*_ (green) and FORM-Fdh_*Ps*_ (orange). Strains were cultivated in N-CgXII, supplemented with 40 g L^−1^ glucose and 60, 120, or 160 mM formamide in a BioLector microcultivation system for 96 h. Values represent means with standard deviations of triplicate cultivations

When these strains were cultivated in N-CgXII supplemented with 60, 120, or 160 mM formamide, comparable effects on the growth rate and maximal biomass concentration were observed (Fig. 7a, b). However, strain FORM-Fdh_*Cg*_ grew faster than the other strains (at 160 mM formamide *µ*_max_ was 13% higher) and at 160 mM formamide showed a lag phase shorter by 40% (Fig. 7b, c). These improved growth characteristics revealed overexpression of native formate dehydrogenase as an effective strategy to alleviate the formate-mediated growth perturbation of *C. glutamicum* with formamide and formamidase.

**Fig. 8:**
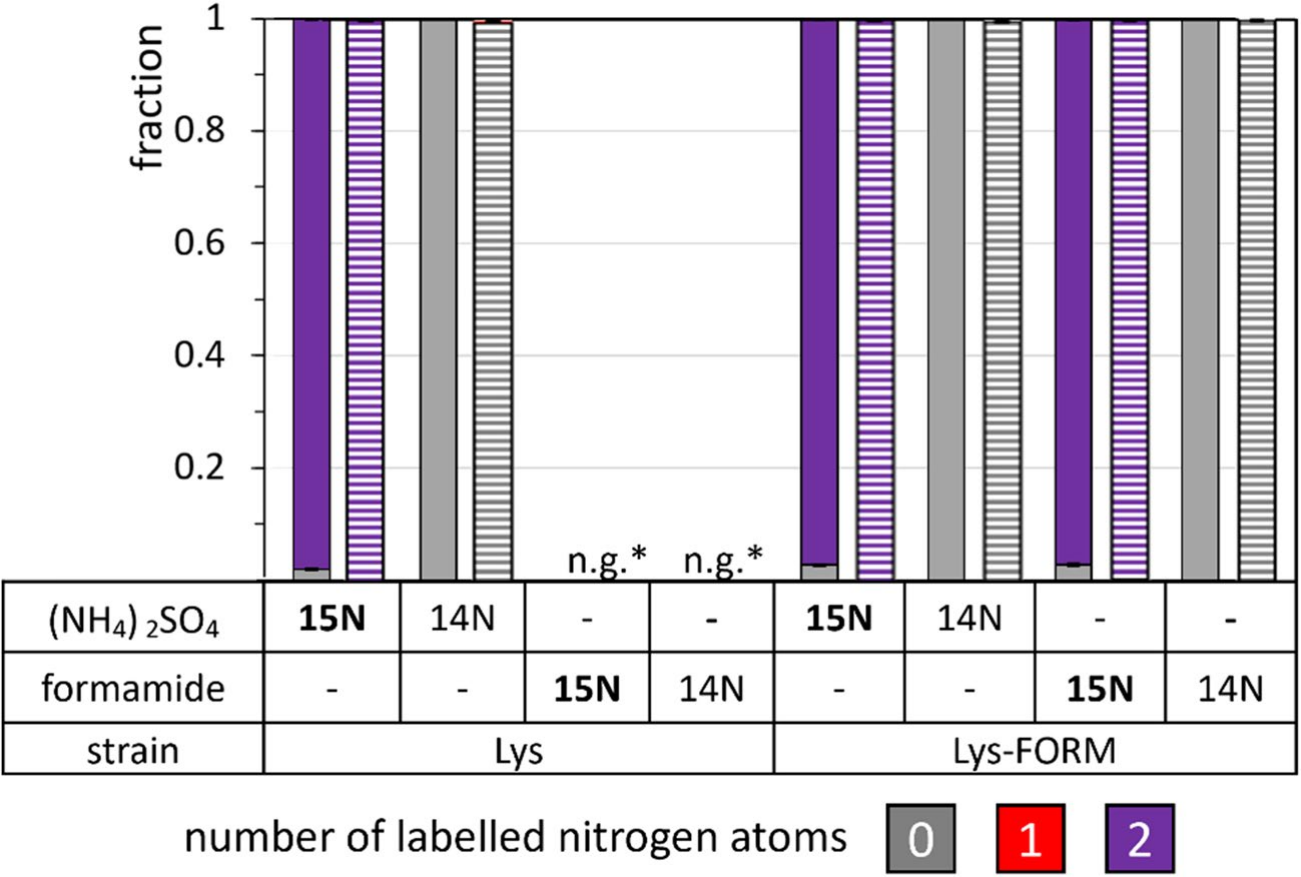
^15^N labelling of lysine in biomass and supernatants of strains Lys and Lys-FORM. Cells were grown in N-CgXII, supplemented with 60 mM unlabeled (14N) or labeled (15N) (NH_4_)_2_SO_4_ or formamide, using 40 g L^−1^ glucose, in a BioLector microcultivation system for 24 h. The patterns depict the fractions of 0 (grey), 1 (red), or 2 (purple) of labeled nitrogen atoms per molecule of lysine in the biomass (filled bars) or the supernatant (dashed bars). Values represent means with standard deviations of triplicate cultivations. *n.g., no growth with formamide

**Fig. 9:**
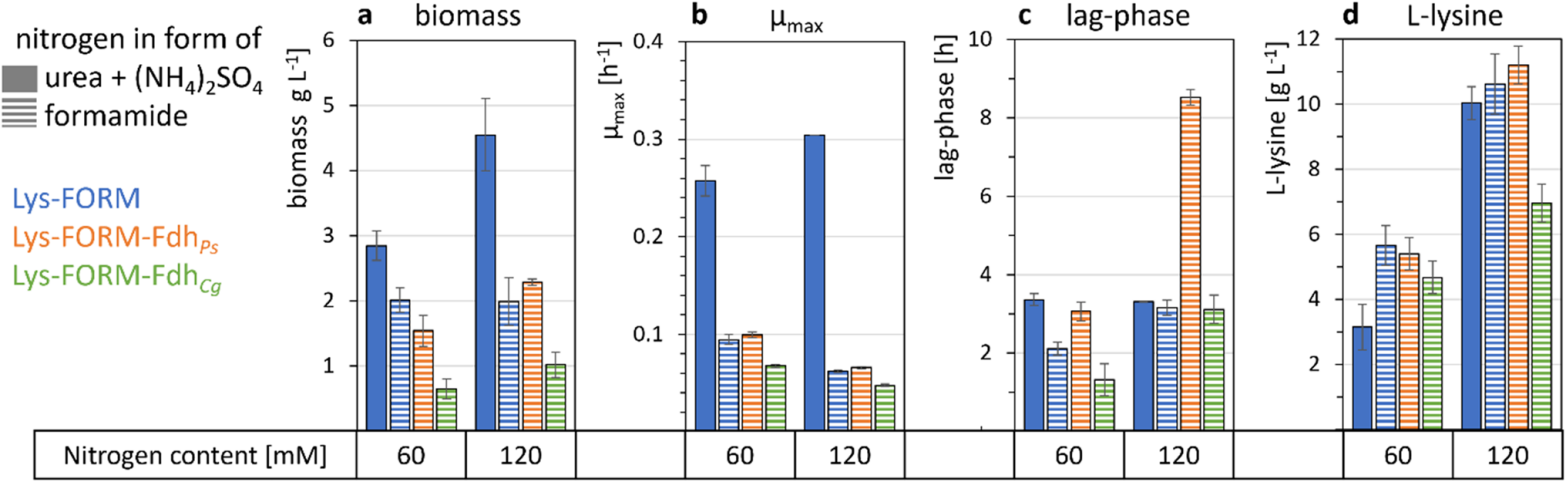
Biomass formation (**a**), maximal growth rates *µ*_max_ (**b**), lag phases (**c**), and L-lysine titers (**d**) for strains Lys-FORM (blue), Fdh-overexpressing Lys-FORM-Fdh_*Ps*_ (orange), and Lys-FORM-Fdh_*Cg*_ (green). Strains were cultivated in N-CgXII, supplemented with 60 or 120 mM nitrogen in form of urea and (NH_4_)_2_SO_4_ (filled bars) or formamide (dashed bars) and 40 g L^−1^ glucose, in a BioLector microcultivation system for 96 h. Values represent means with standard deviations of triplicate cultivations

Therefore, based on the lysine producer *C. glutamicum* GRLys1Δ*sugR*Δ*ldhA* (Pérez-García et al. 2016), referred to as Lys, we constructed the formamidase-positive derivatives Lys-FORM, Lys-FORM-Fdh_*Cg*_, and Lys-FORM-Fdh_*Ps*_. Lysine production by strains Lys and Lys-FORM was compared in a ^15^N-labeling experiment. Strain Lys could not grow with formamide, but when ^15^N-labeled ammonium sulfate was provided, > 99% of nitrogen atoms of lysine in biomass and supernatants were labeled, whereas no labeling was detectable upon cultivation with unlabeled nitrogen sources (Fig. 8). Lysine in the biomass of strain Lys-FORM as well as in the culture supernatants was uniformly labeled from ^15^N-formamide (Fig. 8), demonstrating that formamide was utilized for growth and lysine production. A comparison of lysine production by strains Lys-FORM, Lys-FORM-Fdh_*Cg*_, and Lys-FORM-Fdh_*Ps*_ revealed that Lys-FORM-Fdh_*Cg*_ produced less lysine from 120 mM formamide (6.96 ± 0.57 g L^−1^) than strain Lys-FORM produced in medium with ammonium and urea (10.62 ± 0.93 g L^−1^), while strain Lys-FORM-Fdh_*Ps*_ produced more lysine from 120 mM formamide (11.20 ± 0.90 g L^−1^; Fig. 9d). The lysine titer of Lys-FORM-Fdh_*Ps*_ was not significantly higher than that of strain Lys-FORM and occurred only after a very long lag phase (Fig. 9c, d).

### Formamide-based production of L-glutamate, N-methyl-L-phenylalanine, and dipicolinate

To test if the developed strategy for formamide utilization can be transferred to production of other compounds, production of L-glutamate, N-methyl-L-phenylalanine (NMePhe), and dipicolinate (DPA) was tested with formamide as sole carbon source. Addition of ciprofloxacin at mid-exponential growth was used to elicit glutamate production (Lubitz and Wendisch 2016). This led to the accumulation of 6.51 ± 0.49 g L^−1^ L-glutamate from 60 mM formamide (Table 2). The use of 60 mM nitrogen as urea and (NH_4_)_2_SO_4_ resulted in a lower L-glutamate titer, but a higher biomass concentration (Table 2). Next, NMePhe and DPA producing strains MePhe5* (Kerbs et al. 2021) and Dpa1 (Schwardmann et al. 2022) were equipped with a plasmid for formamidase expression. The resulting strains MePhe5*-FORM and Dpa1-FORM grew in N-CgXII, supplemented with 60 mM formamide as sole nitrogen source (Table 2), though slower and to lower total biomass concentrations (Fig. 1b). N provision in form of formamide instead of urea and (NH_4_)_2_SO_4_ reduced biomass formation by up to 60% (Table 2), while the NMePhe and DPA product titers from formamide surpassed those from urea and (NH_4_)_2_SO_4_ by up to 80% and amounted to 1.68 ± 0.20 g L^−1^ NMePhe, and 0.56 ± 0.01 g L^−1^ DPA, respectively (Table 2).

**Table 2:** Biomass formation, maximal specific growth rates *µ*_max_, product titers and yields of strains FORM, Lys*-*FORM, MePhe5*-FORM, and Dpa1-FORM. Strains were cultivated in N-CgXII, supplemented with 60 mM nitrogen in form of urea and (NH_4_)_2_SO_4_ or formamide and 40 (L-glutamate, L-lysine, and DPA) or 20 g L^−1^ (NMePhe) glucose in shake flasks for 48 (L-glutamate) or 72 h (L-lysine, DPA, and NMePhe). Values represent means with standard deviations of triplicate cultivations. *n.o., growth was not observed as soon as ciprofloxacin was added

Strain	N-source (60 mM N)	Biomass (g L^−1^)	*µ *(h^−1^)	Product	Product titer (g L^−1^)	*Y*_P/X_(g g^−1^)
FORM	Formamide	*n.o	*n.o	L-glutamate	6.51 ± 0.49	2.56 ± 0.41
	Urea + (NH_4_)_2_SO_4_	*n.o	*n.o		2.74 ± 0.19	1.43 ± 0.09
Lys-FORM	Formamide	2.01 ± 0.09	0.095 ± 0.005	L-lysine	5.65 ± 0.61	2.80 ± 0.21
	Urea + (NH_4_)_2_SO_4_	2.85 ± 0.14	0.257 ± 0.015		3.16 ± 0.70	1.10 ± 0.20
MePhe5*-FORM	Formamide	1.40 ± 0.17	0.055 ± 0.002	NMePhe	1.68 ± 0.20	1.20 ± 0.07
	Urea + (NH_4_)_2_SO_4_	3.35 ± 0.17	0.060 ± 0.003		1.40 ± 0.13	0.42 ± 0.02
Dpa1-FORM	Formamide	3.35 ± 0.09	0.076 ± 0.001	DPA	0.56 ± 0.01	0.17 ± 0.01
	Urea + (NH_4_)_2_SO_4_	4.23 ± 0.09	0.047 ± 0.001		0.52 ± 0.01	0.12 ± 0.00

Taken together, formamide supported production of L-glutamate, L-lysine, NMePhe, and DPA and the observed biomass specific product yields (Y_P/X_) were approximately 1.4- to threefold than with the same nitrogen provision from urea and (NH_4_)_2_SO_4_ (Table 2). Thus, the developed strategy was successfully transferred to the production of various nitrogenous compounds, and formamide was revealed as superior amine source regarding the attainable biomass specific product yields.

## Discussion

This study provides the first report on formamide-based growth of *C. glutamicum*. The activity of the synthetic pathway was substantiated by detection of formamidase activity and stable isotope labeling. In co-culture, ammonium released from a formamidase-positive strain supported growth of a formamidase-deficient strain when formamide was the only nitrogen source. Notably, transfer of this metabolic pathway and substitution of regular nitrogen sources for formamide enabled production of various nitrogenous compounds.

Besides, wild-type *C. glutamicum* growing well in the presence of at least 160 mM formamide, i.e., higher than the concentration tolerated by the naturally *amiF* harboring *H. pylori* 26695 (Vliet et al. 2003) and *amiF* expression in *C. glutamicum* strain FORM, led to formamidase activity of 6.0 ± 0.8 U mg^−1^ which was comparable to the donor *H. pylori* (approximately 2.7 to 5 U mg^−1^ total crude extract protein) (Vliet et al. 2003; Bury-Moné et al. 2004). This activity exceeded that reported for heterologous overexpression of the *amiF* gene in *B. subtilis* (1.2 U mg^−1^) (Guo et al. 2020a, b).

The release of NH_4_^+^ by *H. pylori* in its natural acidic environment may be disadvantageous for other organisms by decoupling the transmembrane pH gradient (Kleiner 1981). Nevertheless, formamide cleavage by strain FORM provided sufficient ammonium to support growth of a co-cultured formamidase-deficient *C. glutamicum* strain. While the uptake system for formamide in *C. glutamicum* is not known, ammonium uptake is mediated by the membrane potential-dependent ammonium transporters AmtA and AmtB (Siewe et al. 1998; Jakoby et al. 2000). A similar observation was made in co-cultures of *E. coli* with one of the cognate amino acids arginine or glutamate serving as sole nitrogen source and leakage of ammonium enabling growth of the co-cultured competitor strain (Wang et al. 2016). However, unwanted background growth by the parental *E. coli* strain with the provided amino acids as sole nitrogen source was possible (Wang et al. 2016), but was not observed here as *C. glutamicum* WT-EV showed no detectable growth with formamide. Thus, formamide utilization as alternative nitrogen source is suited to maintain synthetic consortia with strict mandatory dependency.

The controlled feeding of formamide to a co-culture consisting of formamidase-positive and -negative strains grown with either ammonium and/or urea will favor the formamidase-positive strains (compare Fig. 1a, b), while the provision of formamide as sole nitrogen source will favor formamidase-negative strains (compare Fig. 6) to regulate co-culture dynamics. This approach may complement the strategic inoculation to regulate co-cultivation dynamics as shown for *C. glutamicum* strains utilizing different carbon sources for production of riboflavin in co-culture (Pérez-García et al. 2021). Alternatively, the trait of formamide utilization may be used to design mutualistic synthetic consortia such as shown for co-culturing *E. coli* with *C. glutamicum* for production of cadaverine and L-pipecolic acid from starch (Sgobba et al. 2018a, b). In this context, the choice of a non-nitrogenous product might be preferable to prevent nitrogen limitation perturbing production or the process design would require accurate fine-tuning for balanced and sufficient nitrogen availability.

Previously, anticontamination systems for naturally formamidase deficient *E. coli* and *B. subtilis* strains have been engineered by heterologous expression of *amiF* and use of formamide as sole nitrogen source (Guo et al. 2020a, b; Ou et al. 2019). This type of contamination-free cultivation under non-sterile conditions can also be envisioned for *C. glutamicum* to avoid of time-, and cost-, and resource-intensive sterilization processes and antibiotic addition. However, the ammonium leakage observed for *C. glutamicum* strain FORM may pose a problem as described for the use of phosphite as sole phosphorus source when the gene *ptxD* for phosphite dehydrogenase from *Pseudomonas stutzeri* was expressed (Guo et al. 2020a, b). Hence, the *amiF*/formamide system alone cannot ensure contamination-free non-sterile cultivation. However, the combined use of the *amiF*/formamide and *ptxD*/phosphite systems, as employed for non-sterile cultivation of *E. coli* and *B. subtilis* strains (Guo et al. 2020a, b; Ou et al. 2019), may be required to avoid contaminations.

Formate generated by formamidase from formamide is inhibiting growth to a much larger extent than the other product of formamidase, NH_4_^+^, that is growth inhibitory only at very high extracellular concentrations, supposedly due to elevated ionic strength or osmolarity (Müller et al. 2006). Formate tolerance was suggested to be connected to the activity level of formate oxidizing formate dehydrogenase (Cotton et al. 2020). *C. glutamicum* naturally possesses formate dehydrogenase FdhF (encoded by cg0618) that reduces growth retardation due to 100 mM extracellular formate (Witthoff et al. 2012). Here, we showed that overexpression of genes for native NAD-dependent FdhF as well as NADP-dependent *Pseudomonas* Fdh (Calzadiaz-Ramirez et al. 2020) alleviated growth inhibition by formate generated in the AmiF reaction. However, under the chosen conditions, provision of NADPH by Fdh_*Ps*_ instead of NADH by native FdhF did not improve lysine production, a biosynthesis pathway requiring 4 NADPH molecules per lysine molecule. Such an advantage was only seen when the gene for the native Fdh of a *C. glutamicum* lysine producer strain was disrupted (Ryabchenko et al. 2020), indicating sufficient formate dehydrogenase activity in wild-type *C. glutamicum*.

To the best of our knowledge, formamide was hitherto only used as xenobiotic nitrogen source for the production of nitrogen-free compounds, namely acetoin and 2,3-butanediol by engineered strains of *B. subtilis* and *K. pneumoniae*, respectively (Guo et al. 2020a, b). For the first time, we demonstrated formamide-based overproduction of four nitrogenous compounds. Notably, while DPA yields were comparable with 60 mM formamide to 60 mM nitrogen in form of urea and (NH_4_)_2_SO_4_, formamide-based production of glutamate, lysine, and NMePhe was superior to production with 60 mM nitrogen in form of urea and (NH_4_)_2_SO_4_. The superior yields from formamide were observed at comparatively low nitrogen concentrations. A balance between the concentrations for carbon and nitrogen sources is not only important to increase titers, but may also change product to by-product ratios as observed with 47 mM nitrogen for NMePhe and DPA production, which led to lower formation of byproducts *N*-methylalanine and lysine, respectively (Kerbs et al. 2021; Schwardmann et al. 2022).

## Supplementary Information

Below is the link to the electronic supplementary material.

Supplementary file1 (PDF 121 KB)

## Data Availability

All data are present in the manuscript and its Supplement.
