# Supplementary material for: Formamide-based production of amines by metabolically engineering Corynebacterium glutamicum
Source: Appl Microbiol Biotechnol. 2023 May 29;107(13):4245–60. doi: 10.1007/s00253-023-12592-3 (PMC10313556; doi:10.1007/s00253-023-12592-3)
Supplement: Supplementary file 1 — Supplementary file1 (PDF 121 KB) [file 253_2023_12592_MOESM1_ESM.pdf]

Supplementary material to Applied Microbiology and Biotechnology

Formamide-based production of amines by metabolically engineering *Corynebacterium glutamicum*

Lynn S. Schwardmann[1], Tong Wu[2], Aron K. Dransfeld[1], Steffen N. Lindner[2], Volker F. Wendisch[1]*

[1]Genetics of Prokaryotes, Faculty of Biology and CeBiTec, Bielefeld University, Universitätsstr. 25, 33615 Bielefeld, Germany; l.schwardmann@uni-bielefeld.de (L.S.S.; ORCID 0009-0009-7345-1164); aron.dransfeld@uni-bielefeld.de (A.K.D.; ORCID 0000-0002-5477-6563)

[2]Charité – Universitätsmedizin Berlin, corporate member of Freie Universität Berlin and Humboldt-Universität zu Berlin, Department of Biochemistry, Charitéplatz 1, 10117 Berlin, Germany, tong.wu@charite.de (T.W.; ORCID 0000-0002-9878-4821), steffen.lindner@charite.de (S.N.L.; ORCID 0000-0003-3226-3043)

***** Correspondence: volker.wendisch@uni-bielefeld.de (V.F.W.); Tel.: +49-521-106-5611; ORCID 0000-0003-3473-0012

Tab. S1 Oligonucleotides used in this work. Overhangs for Gibson assembly are indicated by italics and RBS by bold letters

Name	Sequence (5'-3')	Purpose
amiF-fw	_TAATGGTTCCATGGAATTCGAGCTCGGTACCCGGG_**GAAAGGAGGCCCTTCAG**ATGGG	Amplification of *amiF* from pUC57_*amiF-ptxD*
amiF-rv	_CCAAGCTTGCATGCCTGCAGGTCGACTCTAGAG_TTACTTACCGAAGCGACCGCC	Amplification of *amiF* from pUC57_*amiF-ptxD*
amiF-s	CGCGATCATTATCGATCCACAG	Sequencing of *amiF* for verification
crimson-fw	_GAATTCGAGCTCGGTACCCGGG_**GAAAGGAGGCCCTTCAG**ATGGATAGCACTGAGAACGTCATC	Amplification of *crimsom* from pVWEx1_*crimson*
crimson-rv	_CATGCCTGCAGGTCGACTCTAGAG_CTACTGGAACAGGTGGTGGCG	Amplification of *crimsom* from pVWEx1_*crimson*
amiF-gfp-fw	_CCACCGGCGGTCGCTTCGGTAAGTAACTA_**GAAAGGAGGCCCTTCAG**ATGAGTAAAGG	Amplification of *gfp*$_{UV}$ from pECXT_P_{syn}-*gfp*$_{UV}$
gfp-rv	_CCAAGCTTGCATGCCTGCAGGTCGACT_TTATTTGTAGAGCTCATCCATGCC	Amplification of *gfp*$_{UV}$ from pECXT_P_{syn}-*gfp*$_{UV}$
fdh$_{Cg}$-fw	_CCACCGGCGGTCGCTTCGGTAAGTAA_**CTGAAGGGCCTCCTTTC**ATGACAACCCCTCCAACTGAG	Amplification of *fdh*$_{Cg}$ from genomic DNA of *C.glutamicum* WT
fdh$_{Cg}$-rv	_CCAAGCTTGCATGCCTGCAGGTCGACT_TTATCCGAGCTCGCCCGC	Amplification of *fdh*$_{Cg}$ from genomic DNA of *C.glutamicum* WT
fdh$_{Ps}$-fw	_CCCAACCACCGGCGGTCGCTTCGGTAAGTAACT_**GAAAGGAGGCCCTTCAG**ATGCATCATCACC	Amplification of *fdh*$_{Ps}$ from pZ-ASL-FDH-v9
fdh$_{Ps}$-rv	_CCAAGCTTGCATGCCTGCAGGTCGACT_TTAAACAGCTTTTTTGAATTTAGCAGCTTCTTC	Amplification of *fdh*$_{Ps}$ from pZ-ASL-FDH-v9
gfp-s	GATGGATCCGTTCAACTAGCAGAC	Sequencing of *gfp*$_{UV}$ for verification
pECXT_s-rv	TACTGCCGCCAGGCAAATTC	Sequencing of pECXT_P_{syn} plasmids for verification
pECXT_s-fw	TCAGTGAGCGAGGAAGC	Sequencing of pECXT_P_{syn} plasmids for verification
